# The Effect of Sacubitril/Valsartan on Device Detected Arrhythmias and Electrical Parameters among Dilated Cardiomyopathy Patients with Reduced Ejection Fraction and Implantable Cardioverter Defibrillator

**DOI:** 10.3390/jcm9041111

**Published:** 2020-04-13

**Authors:** Vincenzo Russo, Roberta Bottino, Anna Rago, Andrea Antonio Papa, Biagio Liccardo, Riccardo Proietti, Vincenzo Manna, Paolo Golino, Antonio D’Onofrio, Gerardo Nigro

**Affiliations:** 1 Chair of Cardiology, Department of Translational Medical Sciences, University of Campania “Luigi Vanvitelli”, via Leonardo Bianchi, 80131 Naples, Italy; ro-bottino@hotmail.com (R.B.); 91manvin@gmail.com (V.M.); paolo.golino@unicampania.it (P.G.); gerardo.nigro@unicampania.it (G.N.); 2 Department of Cardiology, Monaldi Hospital, via Leonardo Bianchi 1, 80131 Naples, Italy; anna_rago@alice.it (A.R.); andreaantoniopapa@libero.it (A.A.P.); liccardob@gmail.com (B.L.); antonio.donofrio@ospedalideicolli.it (A.D.); 3 Department of Cardiac, Thoracic, and Vascular Sciences, University of Padua, via Giustiniani 2, 35121 Padua, Italy; riccardo.proietti@unipd.it

**Keywords:** sacubitril/valsartan, tachyarrhythmia, ICD, dilated cardiomyopathy, electrical parameters, atrial fibrillation, shock

## Abstract

Sacubitril/valsartan therapy reduces sudden cardiac death (SCD) among patients with reduced ejection fraction (HFrEF) when compared to guidelines recommended doses of enalapril, however the mechanism is still not clear. There are few, contrasting results about the effect of sacubitril/valsartan on arrhythmias in the clinical context of dilated cardiomyopathy (DCM) and there are no clinical data about its effect on measured implantable cardioverter defibrillator (ICD) electrical parameters, such as atrial/ventricular electrograms sensing and pacing threshold. We conducted a 12 month follow-up observational study in 167 ischemic and nonischemic DCM patients (mean age 68.1 ± 11.6 years; 85% male), with dual-chamber ICD on sacubitril/valsartan treatment, to evaluate the incidence of device detected tachyarrhythmia events, both atrial and ventricular, and the change in measured ICD electrical parameters. We collected data on clinical, electrocardiographic and echocardiographic parameters to find a possible electro-mechanical correlation within results. Our results show that DCM patients with reduced ejection fraction and ICD on sacubitril/valsartan treatment experienced a reduction in both atrial and ventricular arrhythmias incidence and an improvement in ICD electrical atrial parameters. The findings might be explained by the electro-mechanical cardiac reverse remodeling induced by sacubitril/valsartan therapy.

## 1. Introduction

Sacubitril/valsartan therapy demonstrated superiority in reducing the risks of death and hospitalization for heart failure (HF) among patients with reduced ejection fraction (HFrEF) when compared to guideline recommended doses of enalapril; however, no information about the mechanism leading to sudden cardiac death (SCD) was provided in the randomized control trial [1]. There are few and contrasting real-world data about the incidence of device detected tachyarrhythmias in HFrEF patients with implantable cardioverter defibrillators (ICD) treated with sacubitril/valsartan [2,3,4], and its possible direct antiarrhythmic effect is still debated [5,6,7,8,9,10]. Moreover, there are no clinical data about the effect of sacubitril/valsartan therapy on measured ICD electrical parameters, such as atrial/ventricular electrograms sensing and pacing threshold. Therefore, we sought to evaluate the incidence of device-detected tachyarrhythmia events, both atrial and ventricular, and the change in measured ICD electrical parameters among dilated cardiomyopathy (DCM) patients with ICD on sacubitril/valsartan treatment during a 12 month follow-up.

## 2. Materials and Methods

### 2.1. Study Population

From a large cohort of 1237 patients with DCM, both ischemic and non-ischemic, who underwent ICD implantation and were followed from January 2015 to January 2018 at our hospital, we evaluated 765 ICD recipients with DCM, left ventricular ejection fraction (LVEF) ≤40%, functional (New York Heart Association (NYHA) class II—despite optimal medical therapy—who were in need of sacubitril/valsartan therapy according to the current guidelines. Patients with permanent atrial fibrillation (*n*: 57), pacemaker dependency (*n*: 22), subcutaneous ICD (*n*: 123), cardiac resynchronization therapy device (CRT) (*n*: 162), single chamber ICD (*n*: 115), dual chamber ICD—less than one year—(*n*: 60), and prosthetic heart valves (*n*: 45) were excluded from this analysis. Finally, we prospectively enrolled 181 DCM patients with dual chamber ICD in need of sacubitril/valsartan.

### 2.2. Study Protocol

This study was a single-center prospective observational cohort study. The study population underwent medical history examination; physical examination; laboratory evaluation; 12-lead surface electrocardiogram (ECG); 2-dimensional color Doppler echocardiography and device interrogation at enrollment, before sacubitril/valsartan administration, and at six- and 12 months follow-up. The institutional ethics committee approved the protocol. Written, informed consent for participation was provided for all patients.

### 2.3. Drug Administration

The recommended sacubitril/valsartan starting dose was 49/51 mg twice daily. The goal sacubitril/valsartan dose was 97/103 mg twice daily. The starting dose for patients with severe renal impairment or taking low doses of angiotensin converting enzyme inhibitor (ACEI) or angiotensin receptor blocker (ARB) was 24/26 mg twice daily.

### 2.4. Clinical and Laboratory Evaluation

All subjects underwent medical history and physical examination with registration of the NYHA class, as well as a six minute walking test (6MWT) in which a 15 m flat, obstacle-free corridor, was used. The patients were instructed to walk as far as possible, pausing to rest when needed and turning 180° every 15 m within 6 min. Each patient underwent two tests; the first one allowed the subject to familiarize themself with the test. Serum levels of hemoglobin (g/dL), sodium (mmol/L), potassium (mmol/L), creatinine (mg/dL), NTproBNP (pg/mL), and copeptin (CP) (pmol/L) were measured at baseline and at follow-up.

### 2.5. Electrocardiographic Measurements

A 12-lead surface ECG was recorded at a paper speed of 50 mm/s and gain of 10 mm/mV in the supine position. In order to avoid diurnal variations, ECG recordings were generally taken at the same time in the morning (9:00–10:00 a.m.). ECGs were digitally acquired by an optical scanner and then magnified by 400% using Adobe Photoshop software (Adobe Systems Inc., San Jose, CA, USA). 

All electrocardiographic measurements were manually performed by two investigators blinded to the subjects’ clinical status with the use of digitizing computer software. The analysis included three consecutive heart cycles for each ECG lead wherever possible. P wave duration and dispersion, QT and JT dispersion and transmural dispersion of repolarization (TDR) were measured as previously described [11]. Inter-and intra-observer coefficients of variation for ECG parameters less than 5% were considered non-significant. The Bazett’s formula was used to correct the QT/JT interval for the heart rate (QTc = QT/√RR; JTc = JT/√RR).

### 2.6. Echocardiographic Evaluation

A standard ultrasound machine (Vivid 9, GE Medical Systems, Milwaukee, WI, USA) with a 3.5–4 MHz phased-array probe (M3S) was used for gathering information on cardiovascular anatomy and function. All the echocardiographic studies were digitally stored and all the measurements were taken offline by two independent observers who were blinded to the clinical status of the subjects.

Left ventricular (LV) diameter and wall thickness were measured from the two-dimensional targeted M-mode echocardiographic tracings in the parasternal long axis view. Ejection fraction was measured by means of modified Simpson’s biplane method. Each representative value was obtained from the average of three measurements. LV mass was determined and indexed to body surface area. Left atrial size was determined by LA volume, measured using the disk summation algorithm.

Pulsed-wave Doppler examination was performed to obtain peak mitral inflow velocities at early (E) and late (A) diastole and E/A ratio. From the apical 4-chamber view, we performed a pulsed-wave tissue Doppler echocardiography at septal and lateral mitral annulus to assess the E/e’ ratio. Mitral valve regurgitation was quantified as recommended by the European Association of Cardiovascular Imaging [12].

### 2.7. Device Interrogations

During the ICD interrogation the intrinsic P-and R-wave voltage, atrial and ventricular pacing threshold at a pulse duration of 0.4 ms, bipolar pacing leads impedance at 5 V and 0.4 ms, the percentage of atrial and ventricular pacing, the amount of sustained ventricular tachycardia (VT, defined >30 s), ventricular fibrillation (VF), non-sustained ventricular tachycardia (NsVT, defined as ≥4 beats <30 s), sustained atrial tachycardia (AT)/atrial fibrillation (AF) episodes (AT/AF, defined as high atrial rate ≥30 s), and the appearance of appropriate (i.e., shocks and antitachycardia pacing due to VT/VF) or inappropriate (i.e., shocks and antitachycardia pacing due to AT/AF) therapies have been evaluated. The type of tachycardia as defined by the device was confirmed by two independent electrophysiologists blinded to the patients’ clinical status. The ICDs were programmed to minimize unnecessary atrial and right ventricular pacing. Pacing mode was set to DDD with a lower rate of 50 bpm. Algorithms promoting intrinsic rhythm were activated. Three detection zones for VT/VF (VT1 at 150–169 bpm; VT2 at 170–200; VF >200 bpm) were programmed: VT1 was the monitor only zone; the VT2 zone included up to three ATPs and eight shocks; and the VF included only shocks.

### 2.8. Statistical Analysis

The distribution of data was assessed by using both the Kolmorov–Smirnov and the Shapiro–Wilk test. The continuous variables were expressed as mean ± SD or median with interquartiles, as appropriate. The categorical variables were expressed as percentages. The paired Student’s *t*-test/Mann-Whitney test or the chi-square test/Fisher’s exact test were used for continuous and categorical variables, respectively. The differences between mean data over time were determined by means of repeated-measures analysis of variance with Bonferroni’s correction. A two-sided *p* < 0.05 was considered significant for all tests. Analysis was performed using the statistical package SPSS 11.0 software for Windows SPSS Inc. (Chicago, IL, USA).

## 3. Results

### 3.1. Patients Population

From the initial enrolled cohort of 181 DCM patients with dual chamber ICD who were prescribed sacubitril/valsartan according to established recommendations, six patients discontinued sacubitril/valsartan because of hypotension, two because of worsening renal function, two patients died, three patients lost to follow-up and one patient underwent CRT placement during follow-up.

The remaining 167 DCM patients (mean age 68.1 ± 11.6 years; 85% male) with dual-chamber ICD, completed the follow-up and were finally evaluated. The ICD was implanted 757 ± 152 days before the enrollment in the present study. The study population received ACEi or ARB for at least six months before ICD implantation (1137 ± 267 days). Baseline features of the study population are detailed in Table 1.

### 3.2. Clinical and Laboratory Evaluation

Compared with baseline, the NYHA functional class significantly improved and 6-MWT distance significantly increased (257 ± 122 vs. 343 ± 134 m, *p* = 0.001) at six months follow-up. In parallel, a significant reduction in NT-proBNP (427.3 ± 69.3 vs. 380.1 ± 56.3, *p* = 0.02) and CP values (31.8 ± 9.1 vs. 14.22 ± 6.4, *p* = 0.003) was registered at six months follow-up. These values remained stable over the follow-up period. Clinical and laboratory evaluation is listed in Table 2.

### 3.3. Electrocardiographic Evaluation

At the six month follow-up, the study population showed a statistically significant reduction in maximum P wave duration (126 ± 12 vs. 115 ± 15 ms, *p* = 0.03) and P wave dispersion (35 ± 6 vs. 26 ± 5 ms, *p* = 0.02), which remains stable over the follow-up period. A non-significant trend in the reduction of all indexes of heterogeneity of ventricular repolarization (QTc, JTc, TDR) was recorded during follow-up. Results are detailed in Table 3.

### 3.4. Echocardiographic Evaluation

All 167 patients had baseline and follow-up echocardiographic evaluation available for paired analysis. The baseline and follow-up echocardiographic analysis are reflected in Table 4. Following the initiation of sacubitril/valsartan, patients exhibited a significant reduction in both atrial and ventricular volumes, resulting in improved left ventricular eject fraction (LVEF, 28.1 ± 3.2 vs. 33.4 ± 3.1 %, *p* = 0.01) and E/A ratio (1.7 ± 1.2 vs. 0.8 ± 0.8, *p* = 0.003).

### 3.5. ICD Electrical Parameters

At six months follow-up, the P wave amplitude significantly increased (3.2 ± 1.9 vs. 3.4 ± 2.2 mV, *p* = 0.003). The atrial pacing threshold (0.9 ± 0.3 vs. 0.5 ± 0.2 V, *p* = 0.001), the atrial lead impedance (564.3 ± 163.6 vs. 528.2 ± 131.8 Ohm; *p* = 0.04) and the shock impedance (64.8 ± 16.2 vs. 61.2 ± 15.3 Ohm; *p* = 0.01) significantly decreased from baseline and remained stable over the follow-up period. No statistically significant trend in reduction of R wave amplitude (13.4 ± 7.6 vs. 12.9 ± 8.1 mV; *p* = 0.4), ventricular pacing threshold (0.8 ± 0.4 vs. 0.7 ± 0.4 V; *p* = 0.7) and ventricular lead impedance (532.3 ± 170.9 vs. 528.4 ± 176.2 *p* = 0.5) values were recorded over time. The percentage of atrial and ventricular pacing remained stable over the follow-up period (Table 5).

### 3.6. ICD Electrical Parameters

At baseline, sustained VT/FV was recorded in 15 patients and was treated by appropriate and effective shock (*n*: 13) or antitachycardia pacing (ATP, *n*: 2). Inappropriate shocks occurred in 4 patients. Non-sustained VT was recorded in 22 patients and AT/AF in 34 patients. At 12 months follow-up, sustained VT/FV occurred in four patients and was treated in all cases by appropriate and effective shock. No inappropriate shocks were recorded. Non-sustained VT occurred in eight patients; AT/AF in 19 patients. At 12 months follow-up, sustained VT/VF episodes (15 vs. 4, *n*; *p* = 0.03), non-sustained VT (22 vs. 8, *n*; *p* = 0.01), appropriate shocks (13 vs. 3, *n*; *p* = 0.02) and paroxysmal AT/AF episodes (34 vs. 19, *n*; *p*: 0.03) significantly decreased among the study population (Table 6).

## 4. Discussion

There are few real-world data about the clinical performance of sacubitril/valsartan among patients with heart failure and implantable cardioverter defibrillators [2,3,4]. Therefore little is known about the effect of sacubitril/valsartan on device-detected arrhythmias, both ventricular and atrial [2,3,4,5,6,7,8], and there are no reliable data on ICD electrical parameters among patients with heart failure on sacubitril/valsartan treatment.

Our study adds some novel findings on this topic; in particular, we firstly showed a statistically significant reduction in atrial fibrillation episodes on sacubitril/valsartan treatment in DCM patients with reduced ejection fraction and ICD. These data, in contrast with the results of the Paradigm HF Trial [1] and other real-world experiences [6,7,8], may be explained by the electro-mechanical atrial reverse remodeling [13], since significant reduction in atrial volumes, as well an improvement in electrocardiographic indexes of heterogeneity in atrial conduction, were observed in our study population after sacubitril/valsartan treatment. Our findings expand the recently described positive effect of sacubitril/valsartan treatment on atrial volumes [14,15] and among atrial electrical parameters, suggesting the hypothesis of a complete electro-mechanical atrial reverse remodeling.

To the best of our knowledge, this was the first study evaluating the performance of ICD electrical parameters, with a statistically significant improvement in P wave sensing, in atrial pacing threshold and in ventricular shock impedance on sacubitril/valsartan therapy. These results might be explained by a waning course in the level of fibrosis involving the myocardial tissue near the device lead tip. This phenomenon seems to early appear in atrial myocardium among our study population and it might be explained by the significant electro-mechanical atrial reverse remodeling on sacubitril/valsartan treatment [14].

Different from atrial electrical parameters, we found no significant changes in R-wave sensing and in ventricular pacing threshold during the follow-up, probably because the electrical reverse remodeling in the ventricles develops slower than in atria and might be dissociated from the mechanical ventricular reverse remodeling, which is usually earlier [16].

Sacubitril/valsartan simultaneously augments the natriuretic peptide system by inhibiting the enzyme neprilysin and inhibits the renin-angiotensin-aldosterone system by blocking the angiotensin II receptor. This combined action results in multiple beneficial effects; decreasing sympathetic tone, slowing cardiac fibrosis, and inducing the hemodynamic benefits of cardiac reverse remodeling [10,17,18].

Moreover, we showed a statistically significant reduction in the number of patients who experienced VT/VF episodes, both sustained and non-sustained, and appropriate ICD shock events on sacubitril/valsartan treatment at 12 months follow-up. These results may be a consequence of the mechanical and functional reverse ventricular remodeling, since we documented the reduction in ventricular volumes and the improvement in left ventricular function, both systolic and diastolic, confirming the evidences from previous studies [2,15]. However, in contrast with Gonçalves et al. [9], we showed a trend in improvement in electrocardiographic indexes of dispersion of ventricular repolarization, which did not reach the statistical significance. These data suggest the hypothesis that different underlying mechanisms, besides the reduction in the electro-mechanical dispersion of ventricular repolarization, might explain the antiarrhythmic effects of sacubitril/valsartan therapy.

In our analysis, we showed a significant reduction in circulating plasma levels of both NT-proBNP, as previously demonstrated [14,15], and copeptin, a product of pre-pro-arginin-vasopressin (AVP) procession during axonal transport from the hypothalamus to posterior pituitary, used for its stability as a surrogate AVP marker.

The enhanced activity of AVP system, on the one hand, may suggest and predict a worse left ventricular function, with a consequent increased risk of life-threatening arrhythmias; on the other hand, it may have a direct pro-arrhythmogenic effect on myocardium, promoting myocardial fibrosis, and thus enhancing the electrical ventricular vulnerability [19].

A reduction in copeptin serum levels by sacubitril/valsartan therapy might be one of the mechanisms implied in the reduction of arrhythmias burden. Finally, as expected, we found an improvement in patients’ symptoms with a significant decrease in class NYHA level and a better physical performance tested with the six MWTs, strengthening the reliability of sacubitril/valsartan pharmacological and clinical effect.

The present study has some limitations. It is a single-center prospective observational experience with a limited follow-up time, however, no longer follow-up investigating this specific topic is yet available in a real-world setting. There is no control group on ACEI or ARB therapy. However, after the results of the PARADIGM-HF trial [1] it would not be ethical to deprive patients who are in need of pharmacological therapy that has been shown to improve survival. The evaluation of the NYHA class was subjective. The sample size was not large, nevertheless we could reach statistically significant results. However, larger, multicentric and randomized studies are necessary to confirm our preliminary results.

## 5. Conclusions

Dilated cardiomyopathy patients with reduced ejection fraction and implantable cardioverter defibrillators on sacubitril/valsartan treatment experienced a reduction in both atrial and ventricular arrhythmias incidence and an improvement in ICD atrial electrical parameters. The findings might be explained by the electro-mechanical cardiac reverse remodeling on sacubitril/valsartan therapy.

## Figures and Tables

**Table 1:** Shows the baseline characteristics of the study population.

Characteristics	Baseline
Age (years)	68.1 ± 11.6
Male (%)	84.5
Weight (Kg)	77.3 ± 12.9
Body mass index (Kg/m2)	28.5 (21.2–41)
Ischaemic DCM (%)	52.1
Non-ischaemic DCM (%)	47.9
Ejection Fraction (%)	28.1 ± 3.2
Smoke (%)	60
Hypertension (%)	68
Diabetes (%)	41
Dyslipidemia (%)	56
Previous stroke (%)	4
COPD (%)	27
Peripheral artery disease (%)	38
ACE-I or ARB (%)	100
Beta-blocker (%)	98
Ivabradin (%)	10
Calcium Antagonist (%)	4
Amiodarone (%)	10
Sotalol (%)	5
Aldosterone antagonist (%)	90
Loop diuretic (%)	95
Thiazide diuretic (%)	15

DCM: dilated cardiomyopathy; COPD: chronic obstructive pulmonary disease; ACE-I: angiotensin converting enzyme inhibitor; ARB: angiotensin receptor blocker.

**Table 2:** Clinical and laboratory values at baseline and over time.

	Baseline	6 Months	12 Months	*p* *
Systolic blood pressure (SPB)	122 ± 19	118 ± 18	120 ± 20	0.8
Diastolic blood pressure (SPB)	68 ± 11	65 ± 13	67 ± 12	0.8
Heart rate (bpm)	62 ± 4	60 ± 6	61 ± 5	0.8
NYHA Class I (%)	0	15	18	0.01
NYHA Class II (%)	67	75	71	0.4
NYHA Class III (%)	33	10	10	0.04
6-MWT distance (m)	257 ± 122	343 ± 134	338 ± 142	0.001
Hemoglobin, (g/dL)	13.3 ± 1.5	13.2 ± 1.4	13.4 ± 1.3	0.7
Sodium (mmol/L)	141 ± 4	145 ± 6	144 ± 5	0.8
Potassium (mmol/L)	4.3 ± 0.6	4.4 ± 0.5	4.4 ± 0.7	0.8
Creatinine (mg/dL)	1.2 ± 0.4	1.3 ± 0.3	1.2 ± 0.5	0.9
NT-proBNP (pg/mL)	427.3 ± 69.3	380.1 ± 56.3	376.5 ± 62.3	0.02
CP (pmol/L)	31.8 ± 9.1	14.22 ± 6.4	13.18 ± 9.6	0.003

NYHA: New York Heart Association; 6-MWT: six minute walking test; NT-proBNP: N-terminal pro b-type natriuretic peptide; CP: copeptin. *p* *: refers to six months follow-up. The results remain with the same significance level over time.

**Table 3:** Electrocardiographic parameter values at baseline and over time in the overall study population.

	Baseline	6 Months	12 Months	*p* *
Heart Rate (bpm)	62 ± 4	60 ± 6	61 ± 5	0.8
PR duration (ms)	142 ± 17	144 ± 16	143 ± 18	0.7
QRS duration (ms)	121 ± 8	119 ± 11	120 ± 9	0.7
Maximum P wave duration (ms)	126 ± 12	115 ± 15	115 ± 16	0.03
P wave dispersion (ms)	35 ± 6	26 ± 5	25 ± 7	0.02
QTc dispersion (ms)	68 ± 9	65 ± 6	63 ± 7	0.07
JTc dispersion (ms)	53 ± 4	49 ± 5	47 ± 6	0.06
TDR (ms)	36 ± 15	34 ± 11	32 ± 9	0.06

TDR: transmural dispersion of repolarization; *p* * = refers to six months results. The results remain with the same significance level over time.

**Table 4:** Echocardiographic parameter values at baseline and over time in the overall study population.

	Baseline	12 Months	*p*
LVEDV (ml)	226.7 ± 33.7	208.2 ± 80.2	0.02
LVESV (ml)	154.7 ± 24.2	137.1 ± 73.2	0.03
LAVI (ml/m2)	48.2 ± 11.3	38.3 ± 8.1	0.02
RAVI (ml/m2)	34.2 ± 17.1	26.3 ± 13.2	0.02
LV Ejection fraction (%)	28.1 ± 3.2	33.4 ± 3.1	0.01
E/A ratio	1.7 ± 1.2	*0.8 ± 0.8*	0.003
TAPSE (mm)	13 ± 3	*15 ± 7*	0.4
MR 3–4 + (%)	32	24	0.04
PAPs (mmHg)	64 ± 8	40 ± 6	0.001

LVEDV: left ventricular end-diastolic volume; LVESV: left ventricular end-systolic volume; LAVI: left atrial volume index; RAVI: right atria volume index; LV: left ventricle; TAPSE: Tricuspid annular plane systolic excursion; MR: mitral regurgitation; PAPs: systolic pulmonary artery pressure.

**Table 5:** Electrical parameter values at baseline and over time in the overall study population.

	Baseline	6 Months	12 Months	*p* *
P wave amplitude (mV)	3.2 ± 1.9	3.4 ± 2.2	3.6 ± 2.1	0.003
Atrial pacing threshold (V)	0.9 ± 0.3	0.5 ± 0.2	0.5 ± 0.3	0.001
Atrial lead impedance (Ohm)	564.3 ± 163.6	528.2 ± 131.8	525.3 ± 126.7	0.04
R wave amplitude (mV)	13.4 ± 7.6	12.9 ± 8.1	12.7 ± 7.9	0.4
Ventricular pacing threshold (V)	0.8 ± 0.4	0.7 ± 0.4	0.7 ± 0.3	0.7
Ventricular lead impedance (Ohm)	532.3 ± 170.9	528.4 ± 176.2	527.9 ± 173.9	0.5
Shock impedance (Ohm)	64.8 ± 16.2	61.2 ± 15.3	58 ± 11.2	0.01
Atrial pacing percentage (%)	5 ± 2	5 ± 1	4 ± 2	0.8
Ventricular pacing percentage (%)	3 ± 2	3 ± 1	2 ± 1	0.8

*p* * = refers to six months result. The results remain with the same significance level over time.

**Table 6:** ICD detected arrhythmias and therapies at baseline and over time in the overall study population.

	Baseline	12 Months	*p*
Sustained VT/VF (*n*)	15	4	0.03
Non-sustained VT (*n*)	22	8	0.01
Appropriate shock (*n*)	13	3	0.02
Appropriate ATP (*n*)	2	1	0.9
Non-appropriate shock (*n*)	4	0	0.1
Paroxysmal AT/AF (*n*)	34	19	0.03

VT: ventricular tachycardia; VF: ventricular fibrillation; ATP: antitachycardia pacing; AT: atrial tachycardia; AF: atrial fibrillation.
